# Formulation and Structural Optimisation of PVA-Fibre Biopolymer Composites for 3D Printing in Drug Delivery Applications

**DOI:** 10.3390/polym17182502

**Published:** 2025-09-16

**Authors:** Pattaraporn Panraksa, Pensak Jantrawut, Xin Yi Teoh, Krit Sengtakdaed, Ploynapat Pornngam, Tanpong Chaiwarit, Takron Chantadee, Kittisak Jantanasakulwong, Suruk Udomsom, Bin Zhang

**Affiliations:** 1 Department of Pharmaceutical Sciences, Faculty of Pharmacy, Chiang Mai University, Chiang Mai 50200, Thailand; pattaraporn.pan@cmu.ac.th (P.P.); krit.sengtakdaed@gmail.com (K.S.); ploynapat2001@gmail.com (P.P.); tanpong.ch@cmu.ac.th (T.C.); takron.chantadee@cmu.ac.th (T.C.); 2 School of Pharmacy, Monash University Malaysia, Subang Jaya 47500, Selangor Darul Ehsan, Malaysia; txy1807@gmail.com; 3 School of Pharmacy, University College London, London WC1N 1AX, UK; 4 Division of Packaging Technology, School of Agro-Industry, Faculty of Agro-Industry, Chiang Mai University, Chiang Mai 50100, Thailand; jantanasakulwong.k@gmail.com; 5 Biomedical Engineering Institute, Chiang Mai University, Chiang Mai 50200, Thailand; suruk_u@cmu.ac.th; 6 Department of Mechanical and Aerospace Engineering, Brunel University London, Uxbridge UB8 3PH, UK

**Keywords:** hot-melt extrusion, 3D printing, fused deposition modelling, cassava fibre, biopolymer composites, structural optimisation, drug delivery systems

## Abstract

Additive manufacturing using fused deposition modelling (FDM) is increasingly explored for personalised drug delivery, but the lack of suitable biodegradable and printable filaments limits its pharmaceutical application. In this study, we investigated the influence of formulation and structural design on the performance of polyvinyl alcohol (PVA)-based filaments doped with theophylline anhydrous for 3D printing. To address the intrinsic brittleness and poor printability of PVA, cassava pulp-derived fibres—a sustainable and underutilised agricultural by-product—were incorporated together with polyethylene glycol (PEG 400), Eudragit^®^ NE 30 D, and calcium stearate. The addition of fibres modified the mechanical properties of PVA filaments through hydrogen bonding, improving flexibility but increasing surface roughness. This drawback was mitigated by Eudragit^®^ NE 30 D, which enhanced surface smoothness and drug distribution uniformity. The optimised composite formulation (P_10_F_5_E_5_T_5_) was successfully extruded and used to fabricate 3D-printed constructs. Release studies demonstrated that drug release could be modulated by pore geometry and construct thickness: wider pores enabled rapid Fickian diffusion, while narrower pores and thicker constructs shifted release kinetics toward anomalous transport governed by polymer swelling. These findings demonstrate, for the first time, the potential of cassava fibre as a functional additive in pharmaceutical FDM and provide a rational formulation–structure–performance framework for developing sustainable, geometry-tuneable drug delivery systems.

## 1. Introduction

The integration of three-dimensional (3D) printing into pharmaceutical applications has garnered significant interest in recent years, offering new possibilities for personalised medicine. Compared to conventional pharmaceutical manufacturing techniques, 3D printing provides greater flexibility and customisability, enabling precise control over drug formulation, dosage, and release kinetics. This advancement holds great potential for patient-specific treatment, allowing tailored drug delivery systems that address individual therapeutic needs and have the potential to transform pharmaceutical production [1,2] fundamentally.

Among the various 3D printing techniques applied in pharmaceutical research, such as stereolithography (SLA) [3,4], inkjet printing [5,6], selective laser sintering (SLS) [7,8], semi-solid extrusion printing (SSE) [9,10], direct powder extrusion (DPE) [11,12], and fused deposition modelling (FDM) [13,14]—FDM has emerged as one of the most widely studied methods due to its ease of use, cost-effectiveness, minimal material wastage, and compatibility with a wide range of materials [15]. FDM allows for the fabrication of constructs with complex geometries and tuneable microstructures, which can significantly influence drug release behaviour. Nevertheless, challenges remain in optimising material properties and structural design to achieve controlled and predictable drug release. Selecting appropriate materials and modifying the microstructural features of printed constructs are, therefore, crucial strategies for improving drug delivery performance.

Several polymers, including polymethacrylate-based copolymers (Eudragit^®^), polyvinyl caprolactam-polyvinyl acetate-polyethylene glycol graft copolymer (Soluplus^®^), polyethylene oxide (PEO), polylactic acid (PLA), polycaprolactone (PCL), and polyvinyl alcohol (PVA), have been investigated for FDM printing, each exhibiting distinct processing behaviours and drug release profiles [2]. Among these, PVA has received particular attention because of its biocompatibility, biodegradability, and aqueous solubility [16,17]. Importantly, the physicochemical properties of PVA are strongly influenced by its degree of hydrolysis (DH) and molecular weight (MW), which together determine its crystallinity, melting temperature, solubility, mechanical properties, and processing behaviour [18,19]. Fully hydrolysed PVA (DH > 98.5%) is highly crystalline, rigid, and dissolves slowly in aqueous media. These features provide strong structural strength but also hinder melt processability, making filament extrusion more difficult and challenging. In contrast, partially hydrolysed PVA grades (DH ~ 76–89%) display lower crystallinity and contain more amorphous regions, resulting in enhanced chain mobility and reduced brittleness. Such characteristics not only promote smoother melt flow during hot-melt extrusion (HME) but also yield filaments with greater flexibility and resilience, while offering a more favourable environment for drug dispersion [17,20,21,22,23]. Among partially hydrolysed PVA grades specifically designed for HME, the two most widely used are PVA 3-82 and PVA 4-88. The lower hydrolysis grade (PVA 3-82, DH ~ 80–82%) exhibits stronger interactions with hydrophobic drugs, making it particularly effective as a precipitation inhibitor for poorly soluble active pharmaceutical ingredients (APIs). By comparison, the higher hydrolysis grade (PVA 4-88, DH ~ 85–89%) offers broader applicability, including compatibility with high-melting-point APIs that are otherwise unsuitable for HME [24]. Considering its favourable balance of thermoplasticity, processability, and broad API compatibility, PVA 4-88 was selected in this study as the base polymer for filament fabrication. Despite the advantages mentioned, the use of neat PVA in HME and FDM remains challenging, as it requires high torque, elevated melting temperatures, and often produces brittle filaments prone to breakage during feeding and printing. Moreover, while its high water solubility is advantageous for fast-dissolving drug delivery systems, it may limit its applicability in sustained-release formulations [25].

To overcome the limitations of neat PVA, several modification strategies, such as plasticiser addition, polymer blending, and natural fibre incorporation, have been investigated. Among these, natural fibres stand out as not only sustainable and biodegradable additives but also mechanical modifiers that can improve filament properties, feedability, and drug release performance [26,27,28]. To date, previous studies have examined fibres from agricultural residues, such as flax [29], bamboo [30], sugarcane [31], and hemp hurd [32], but these have been used mainly with polyesters (e.g., PLA, PCL) in packaging or engineering contexts to improve stiffness or thermal stability. Their role in pharmaceutical 3D printing applications, particularly in combination with hydrophilic polymers like PVA, remains underexplored. This gap highlights the need to explore alternative and underutilised fibre sources with potential for developing sustainable PVA-based materials for 3D-printed drug delivery systems. One such source is cassava pulp waste, a by-product of cassava (*Manihot esculenta* Crantz.) starch processing, which is generated in large quantities—over 7 million tonnes annually in Thailand [33]. Improper disposal of this biomass poses serious environmental concerns, including methane emissions, water contamination, and air pollution from open burning in Thailand and Southeast Asia regions [34,35]. Repurposing cassava pulp waste, therefore, not only mitigates environmental impacts but also introduces a new biodegradable and functional excipient for pharmaceutical use.

In this study, cassava fibres were incorporated into PVA-based filaments to produce drug-loaded composites suitable for FDM 3D printing. This work builds on our previous study [36] using powder melt extrusion (PME) of cassava–PVA constructs with indomethacin, but overcomes PME’s limitations in excipient incorporation and drug loading capacity. Here, a two-step process—HME followed by FDM—was employed, offering greater flexibility in formulation and enabling the addition of functional excipients such as Eudragit^®^ and metal stearates to enhance printability, flowability, and drug content uniformity. Theophylline (THP), a model drug with a narrow therapeutic index, was selected to assess drug delivery performance. We further investigated the effect of structural design parameters, including pore width and number of printed layers, on release behaviour. While prior work has shown that geometry can modulate release in hydrophobic polymer systems [37], its applicability in hydrophilic matrices remains unclear. Addressing this gap, the present study explores how formulation, fibre incorporation, and geometry collectively influence release from hydrophilic PVA composites. This integrated optimisation of material and architecture offers a promising strategy for developing sustainable and customisable drug delivery systems using FDM.

## 2. Experiment

The study followed three main stages: material preparation, fabrication, and characterisation. Short fibres were extracted from cassava pulp waste through soaking, enzymatic starch removal, alkaline treatment, drying, and sieving, then blended with polyvinyl alcohol (PVA), plasticisers, functional excipients, and theophylline to produce composite filaments. These filaments were extruded and systematically characterised through morphological and mechanical testing, FTIR spectroscopy, thermal stability analysis, and drug content determination. The drug-loaded filaments were subsequently employed to 3D-print constructs with varied geometries, which further characterised by microstructural evaluation and in vitro release studies to examine formulation–structure–performance relationships. Statistical analysis was performed to validate the findings.

### 2.1. Materials

The materials used in this study, their type, origin, and roles in the pharmaceutical formulation are summarised in Table 1. Short fibre preparation was supported by the Faculty of Agro-Industry, Chiang Mai University. Cassava pulp waste (Premier Quality Starch PCL, Mukdahan, Thailand), a by-product of starch production, was soaked overnight to promote disaggregation and facilitate subsequent treatment. The pulp was then dried at 70 °C for 5 h and treated with α-amylase (20 IU/mL) at 90 °C for 20 min after pH adjustment (6.5–7.0) to remove residual starch, confirmed by iodine testing. The starch-free pulp was filtered, dried, and analysed for holocellulose, lignin, and starch content. Next, 100 g of pulp was chemically treated with 12% NaOH (1:20 ratio) at 85 ± 5 °C for 3 h under stirring to remove hemicellulose and lignin. After washing to neutral pH, the fibres were dried again at 70 °C for 5 h. The final yield was 37.84% (dry weight), consisting of 46.06% cellulose, 1.24% starch, and 5.40% lignin. To ensure consistency in blending and extrusion, the fibres were sieved through a No. 325 mesh, yielding an average particle size of ~45 µm.

All salts used for buffer preparation were purchased from commercial suppliers: sodium chloride and sodium phosphate dibasic dihydrate from RCI Labscan Limited (Bangkok, Thailand), potassium chloride from KEMAUS Chemicals (New South Wales, Australia), and potassium phosphate monobasic from Daejung Chemicals & Metals Co., Ltd. (Gyeonggi, Republic of Korea).

### 2.2. Procedure

#### 2.2.1. Preparation of PVA-Based Composite Filaments

To develop and optimise PVA-cassava fibre composite filaments, various plasticisers, short fibres, additional excipients, and a model drug (THP) were incorporated to enhance filament flexibility, mechanical strength, printability, and drug release characteristics. The fabrication process involved three main steps: plasticisation, fibre incorporation, and drug loading, each systematically optimised to achieve the desired properties for FDM 3D printing. Initially, four plasticisers: mannitol (M), glycerine (G), sorbitol (S), and PEG400 (P), were evaluated at three concentrations (7.5%, 10.0%, and 12.5% *w*/*w*) to identify the most suitable plasticiser and level for maximising filament flexibility while maintaining mechanical stability. The compositions of these formulations are listed in Table 2.

**Figure 1:**
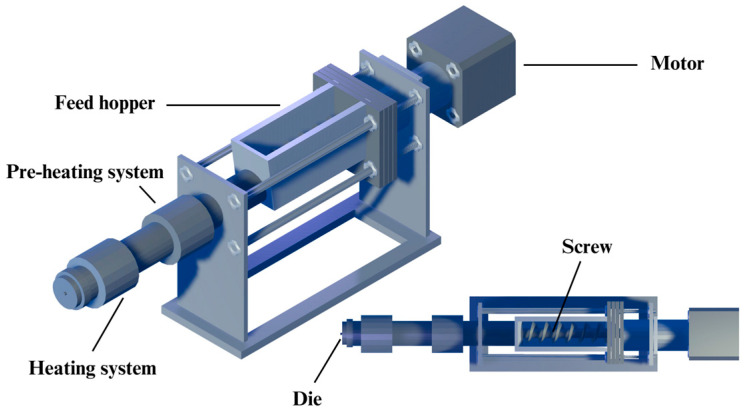
Schematic illustration of a single screw hot-melt extruder.

The PVA–plasticiser blends were prepared by geometric dilution for 10 min, followed by drying in a hot air oven at 60 ± 2 °C for at least 12 h to remove residual moisture. After drying, the mixtures were processed into filaments using a custom-designed, single-screw, hot-melt extruder (developed by the Biomedical Engineering Institute, Chiang Mai University), as illustrated in Figure 1. The extrusion parameters, including pre-heat temperature, extrusion temperature, screw speed, and conveyor belt speed, were carefully optimised for each formulation to ensure smooth extrusion and consistent filament diameter (1.75 mm).

Following plasticiser optimisation, cassava pulp-derived short fibres (5.0% *w*/*w*) were incorporated into the PVA-PEG400 (P_10_) formulation using a geometric dilution method to minimise fibre agglomeration and ensure homogeneous dispersion, with the aim of investigating their effects on filament characteristics and drug release behaviour. A cassava fibre content of 5.0% *w*/*w* was selected based on preliminary screening, as it offered a balance between mechanical enhancement and printability. Lower fibre concentrations did not produce significant changes in mechanical properties, while higher contents resulted in increased surface roughness and void formation, negatively affecting filament extrudability and 3D printability.

To further optimise filament formulation, Eudragit^®^ NE 30 D (5.0% *w*/*w*) was incorporated into selected formulations to assess its role in enhancing filament cohesion. Theophylline (5.0% *w*/*w*) was used as a model drug, and calcium stearate (1.0% *w*/*w*) was added to improve powder flow and extrusion stability, especially in fibre-loaded and drug-containing formulations (Table 3).

The same mixing, drying, and extrusion process was applied to these composite formulations as described earlier. Optimisation of extrusion parameters for fibre-containing formulations was critical to ensure consistent filament quality and avoid defects such as air bubbles or irregularities. The optimised parameters for all formulations are summarised in Table 4.

#### 2.2.2. Design and Fabrication of 3D-Printed Constructs

The design and fabrication of the 3D-printed constructs in this study were carefully tailored to investigate the effects of microstructural features on drug release behaviour, building on our previous work [37], which demonstrated that variations in parameters such as pore width, pore length, pore shape, and filament intersection angles significantly influenced drug dissolution profiles. Expanding on that study, the present work focused specifically on pore width and layer number to further explore how these structural factors impact drug release kinetics in hydrophilic-based composites.

To assess the impact of pore width, a series of cuboid lattice structures was designed with a fixed filament intersection angle of 90° between adjacent layers, consistent with the approach established in our earlier study. The pore width (*d_xy_*), defined as the distance between adjacent extruded filaments within the same layer, was systematically varied from 0.4 to 2.1 mm, representing a gradient from low to high porosity. Specifically, four key pore widths were selected for detailed evaluation: 0.4 mm (low porosity), 0.6 mm (moderate porosity), 1.2 mm (high porosity), and 2.1 mm (very high porosity). For this part of the study, the number of layers was fixed at eight to ensure consistency and isolate the influence of pore width on drug release behaviour. This setup allowed for a controlled investigation of how increasing porosity affects drug release rates by modifying the available surface area and diffusion pathways.

**Figure 2:**
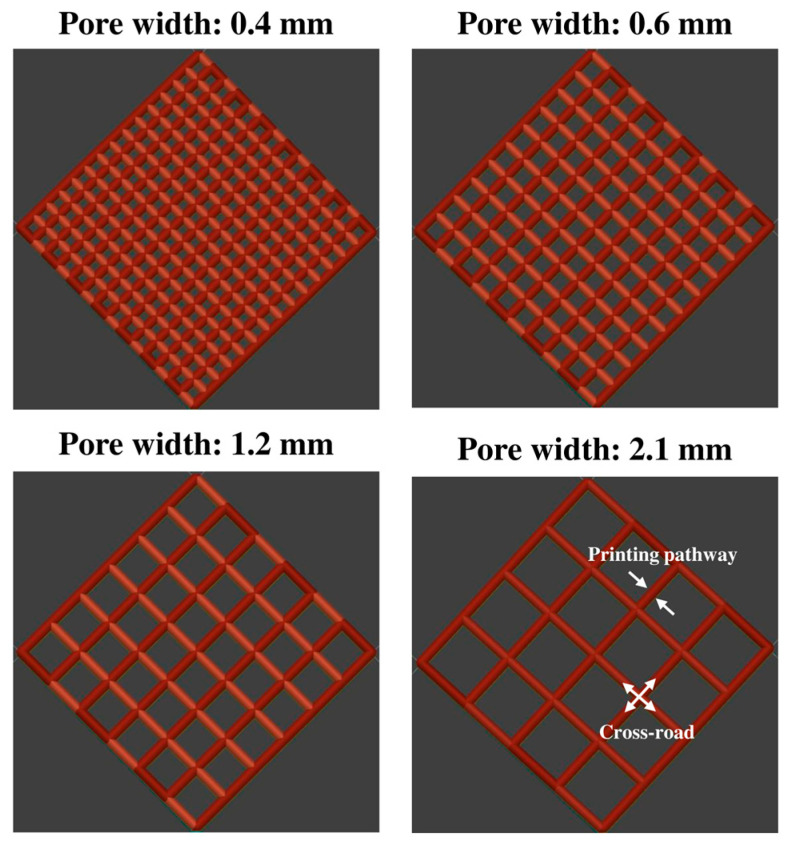
Illustration of the examples of the CAD designs of porous (with pore widths of 0.4, 0.6, 1.2, and 2.1 mm).

In addition to pore width, the effect of construct thickness, which is determined by the number of stacked layers, was also investigated. To achieve this, a second set of lattice constructs was printed with fixed pore widths of 0.6 mm and 2.1 mm while varying the number of layers from 2 to 12. The layer number directly influenced the pore length (*L*) in the Z direction, thereby modifying the overall thickness of the construct. By increasing the number of layers, the study aimed to determine whether the added structural complexity facilitated or hindered drug release. This approach extends the methodology from our previous work, adapting the investigation of pore length to assess layer-induced thickness variations in a hydrophilic polymer-fibre system. The CAD parameters of the different designs of the 3D constructs used in the drug release studies are summarised in Table 5 and visually represented in Figure 2.

All 3D constructs were fabricated using a low-cost FDM 3D printer (Prusa MINI+, Prusa Research a.s., Prague, Czech Republic), equipped with a 400-µm nozzle. The nozzle temperature was maintained at 200 °C, while the print bed was heated to 60 °C to promote adhesion between the first layer and the build platform. Printing speed and extrusion rate were carefully controlled at 20 mm/s and 0.125 mm^3^/mm to ensure consistent deposition across all designs. The 3D printer’s stepper motors operated with a resolution of 0.001 mm per step and a step angle of 1.8°. The G-code for printing was generated using MATLAB 2019b (MathWorks, Natick, MA, USA), enabling precise control over the extrusion paths and construct geometries.

### 2.3. Characterisation

#### 2.3.1. Morphological Analysis of Filaments

The morphological characteristics of the filaments, including surface and cross-sectional characteristics, were analysed using a benchtop scanning electron microscope (SEM) (JCM-7000 NeoScope™, JEOL Ltd., Tokyo, Japan). For SEM imaging, the filaments were first mounted onto aluminium stubs using conductive double-sided carbon tape (Nisshin EM Co., Ltd., Tokyo, Japan). The mounted samples were then coated with a thin layer of gold using an automated sputter coater (JEOL Smart Coater, JEOL Ltd., Tokyo, Japan). Imaging was performed in high-vacuum mode using a secondary electron (SE) detector at an acceleration voltage of 15.0 kV. Micrographs were captured at magnifications of 50× for cross-sectional views and 100× for surface morphology. Additionally, the physical appearance of the filaments was visually evaluated using digital images captured with both a mobile phone and a portable digital USB microscope. Images captured with the mobile phone (Apple iPhone 13, equipped with a dual 12-megapixel camera system) were standardised by positioning the phone at a fixed distance of 20.0 cm from the samples and images were taken at 5× digital zoom. For microscope imaging, a portable USB microscope (Yao, Shenzhen, China), equipped with eight LED lights and a high-definition CMOS sensor, was used. The microscope was positioned at a fixed distance of 3.0 cm from the samples, and images were acquired via camera software for Windows 10 version 2025.2505.2.0 (Microsoft Corporation, Redmond, WA, USA) at a resolution of 640 pixels.

#### 2.3.2. Mechanical Properties

The tensile tests of the filaments were measured using a texture analyser (Stable Micro Systems, Surrey, UK) with a maximum load of 50 kN. The experiment was carried out at a temperature of 25 °C with a crosshead speed of 2 mm/min. The machine has built-in TEConnect 2022 software version 8.0 that allows measurement data to be controlled, monitored, and recorded. The top and bottom jigs grip and secure the sample tightly. During the test, the crosshead continues to move until a distance of 5 mm is reached. The TEConnect software records test data, including force and grip displacement. The filament length used for mechanical testing was approximately 50 mm. The sample diameter was measured at five sections of the specimens, and the average value was used for data analysis. Three samples were measured for each type of filament, and average values were determined.

#### 2.3.3. Fourier Transform Infrared (FTIR) Spectroscopy Analysis

Fourier transform infrared (FTIR) spectroscopy was employed to analyse the chemical structure of all filaments. Infrared spectra were collected using the INVENIO^®^ FT-IR spectrometer (Bruker Corporation, Billerica, MA, USA) in attenuated total reflectance (ATR) mode. Measurements were performed within the spectral wavenumber range of 4000–400 cm^−1^, at a resolution of 4 cm^−1^ with 32 scans per sample. Spectra of reference material, PVA powder, was also recorded for comparison.

#### 2.3.4. Thermal Stability Analysis

The thermal stability of filaments was tested using thermogravimetric analysis (TGA) and differential scanning calorimetry (DSC). Filaments were sliced into thin layers using a blade before being sampled in the sample pan. In TGA analysis, samples were loaded into aluminium cups and heated using Discovery TGA (TA Instruments, New Castle, DE, USA) from 40 to 400 °C at 20 °C/min under 25 mL/min nitrogen purge. DSC analysis was conducted using TA Discovery DSC 2500 (TA Instruments, New Castle, DE, USA). Approximately 3–5 mg of the samples was weighed and sealed in an aluminium pan with a pinhole lid. Samples were heated using a modulated heating mode at 2 °C/min from 0 to 280 °C under 50 mL/min nitrogen purge. All data were analysed using TRIOS software version 5.7.2.101 (TA Instruments, New Castle, DE, USA).

#### 2.3.5. Drug Loading Content Analysis for Theophylline-Loaded Filaments

The drug loading of THP in the developed filaments was determined using a UV spectrophotometer (UV-2600i, Shimadzu Corporation, Tokyo, Japan) with an absorbance measurement at a wavelength of 271 nm. Filament samples were randomly selected from various sections (both ends and centre) of the long filament, with each sample weighing precisely 100 ± 5 mg, equivalent to approximately 5 mg of THP. These samples were dissolved in deionised water for 2 h or until fully dissolved and subsequently diluted 50-fold with deionised water to ensure optimal analyte concentration. The diluted samples were then filtered using a 0.45 µm nylon syringe filter (Labfil^®^, Hangzhou, China) to remove any particulates. To determine the THP concentration, the absorbance of the samples was measured and compared to a pre-established standard curve (*y* = 0.0513*x* + 0.0243, R^2^ = 0.9986), where *y* represents the absorbance and *x* is the concentration of THP in µg/mL. Each sample was analysed in triplicate, and the drug content was reported as the recovery percentage relative to the theoretical content. Results were expressed as mean ± standard deviation (% recovery). The UV-Vis method was validated for linearity, accuracy, and precision prior to analysis. Intra-day (*n* = 3) and inter-day (*n* = 3) reproducibility tests yielded relative standard deviation (RSD) values of 1.07% and 1.46%, respectively, both within the acceptance criterion of <2%, confirming the robustness and reliability of the method for drug loading assessment.

#### 2.3.6. Microstructural Characterisation

The microstructural characteristics of the 3D CAD constructs were evaluated to assess their geometric accuracy, porosity, and surface morphology. The solid volume ($V_{solid}$), and surface (${SA}_{solid}$) of the 3D geometry models were obtained using the CAD software SolidWorks 2022. The surface area-to-volume ratio (SA/V) was calculated using:

(1) $$SA/V=\frac{{SA}_{solid}}{V_{solid}}$$

The surface morphology of the printed constructs was examined using a digital portable USB microscope (Yao, Shenzhen, China) equipped with eight LED lights and a high-definition CMOS sensor. Images were acquired via camera software for Windows 10 (Microsoft Corporation, Redmond, WA, USA) at a resolution of 640 pixels. The microscope was positioned at a fixed distance of 10.0 cm from the samples to ensure consistent imaging conditions. These images were used to assess filament fusion, surface roughness, and the presence of defects in the printed constructs.

#### 2.3.7. In Vitro Drug Release Testing

The in vitro drug release profiles of the 3D-printed constructs were evaluated using a modified pharmaceutical dissolution test. THP-loaded constructs were placed in 20 mL of phosphate-buffered saline (PBS, pH 7.4) and agitated at 100 rpm at 37 °C in a benchtop shaking incubator (NB-205, N-BIOTEK, Gyeonggi, Republic of Korea) to simulate physiological conditions. The composition of PBS was as follows: sodium chloride 8 g/L (0.137 M), potassium chloride 0.2 g/L (0.0027 M), sodium phosphate dibasic 1.44 g/L (0.01 M), and potassium phosphate monobasic 0.245 g/L (0.0018 M) [38]. The sink condition was maintained throughout the drug release period to prevent saturation effects. At predetermined time intervals, 3-mL samples of the dissolution medium were withdrawn and replaced with an equal volume of fresh PBS. The concentration of the released drug was quantified using a UV-Vis spectrophotometer (UV-2600i, Shimadzu Corporation, Kyoto, Japan) at a wavelength of 272.0 nm. The absorbance values were converted to drug concentration using the standard calibration curve: *y* = 0.0572*x* − 0.0108 (R^2^ = 0.9996), where *y* represents the absorbance, and *x* corresponds to the drug concentration in µg/mL. Drug quantification during release testing was reproducible, as confirmed by a validated UV-Vis method. Intra-day and inter-day RSD values were 1.12% and 1.35%, respectively, both within the acceptance criterion of <2%, confirming method robustness for monitoring drug release kinetics.

Each drug release test was performed in triplicate. To analyse the drug release kinetics, the mean dissolution time (MDT) was calculated as follows [39,40]:

(2) $$MDT=\frac{\sum_{j=0}^{n}t_{j^{*}}∆M_{j^{*}}}{\sum_{j=1}^{n}∆M_{j^{*}}}$$

where j is the sample number, n is the number of dissolution time points, $t_{j^{*}}\;$ is the midpoint time between consecutive sample times $t_{j}$ and $t_{j-1}$, and $∆M_{j^{*}}$ is the amount of drug released between $t_{j}$ and $t_{j-1}$.

To further analyse the release mechanism, the experimental data were fitted to the Higuchi and Korsmeyer–Peppas models, as shown below:

(3) $$\mathrm{Higuchi}\;\mathrm{model}:\;\;\;\;\;Q_{t}=\;k_{H}\cdot t^{1/2}$$

(4) $$\mathrm{Korsmeyer}–\mathrm{Peppas}\;\mathrm{model}:\;\;\;\;\;\frac{Q_{t}}{Q_{\infty }}=k_{K}\cdot t^{n}$$

where $Q_{t}$ is the amount of drug released at time t, $\frac{Q_{t}}{Q_{\infty }}$ represents the fraction of drug released at time t, $k_{H}$ and $k_{K}$ are the release rate constants for the Higuchi and Korsmeyer–Peppas model, respectively, and n is the release exponent that indicates the release mechanism.

#### 2.3.8. Statistical Analysis

In this study, statistical analyses were conducted using SPSS Statistics software (version 17.0, SPSS Inc., Chicago, IL, USA). Data are presented as mean ± standard deviation (SD) with a sample size of *n* = 3 unless otherwise specified. A one-way analysis of variance (ANOVA) was performed to determine statistical differences among experimental groups. Statistical significance was set at *p* < 0.05.

## 3. Results and Discussion

### 3.1. Morphological Characteristics of PVA-Based Composite Filaments

The morphological properties of PVA-based composite filaments were evaluated visually and using SEM to understand the influence of plasticisers, short fibres, and additional polymers or THP drug loading on their extrusion performance, as well as their structural and surface characteristics. These analyses provide critical insights into how compositional and processing parameters influenced filament quality, flexibility, and surface texture.

**Figure 3:**
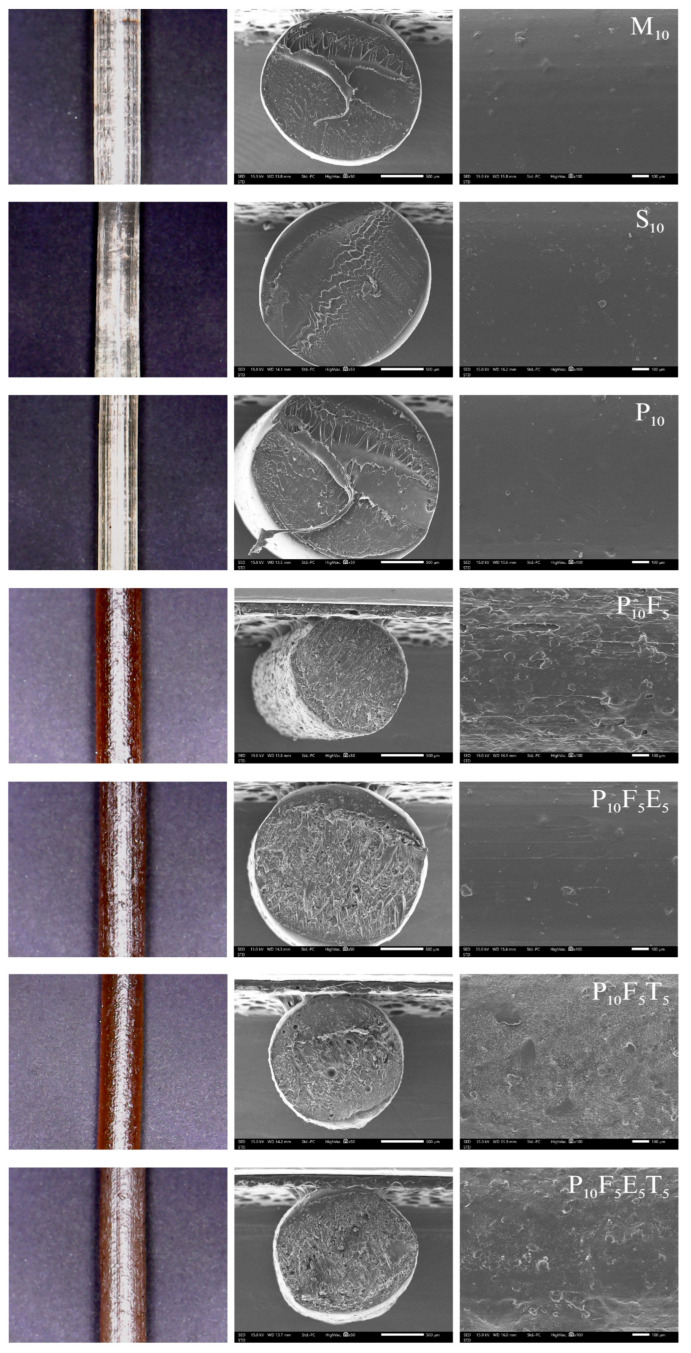
Mini-microscope images (**left**), cross-sectional (**middle**), and surface (**right**) SEM micrographs of PVA-based filaments.

The choice of plasticiser significantly influenced the extrudability, flexibility, and stability of the filaments. Among the tested plasticisers, mannitol (M), glycerine (G), sorbitol (S), and PEG400 (P), mannitol, sorbitol, and PEG400 were effective in facilitating successful filament formation via hot-melt extrusion (HME). As observed in Figure 3 and Figure S1, the M_10_, S_10_, and P_10_ formulations produced clear, light-yellow filaments with smooth surfaces, suggesting uniform plasticiser distribution and adequate structural integrity. SEM micrographs confirmed the homogeneity of these filaments, showing smooth, void-free cross-sections and surfaces. Notably, P_10_ filaments exhibited a slightly smoother surface compared to S_10_ and M_10_, which indicates better homogeneity and potential for improved feedability during FDM 3D printing. The success of mannitol and sorbitol can be attributed to their polyhydric alcohol structures, which interact effectively with the hydroxyl groups of PVA to balance chain mobility and crystallinity. Similarly, PEG400, a polymeric plasticiser, introduced flexibility while maintaining mechanical integrity due to its longer molecular chain. However, formulations with glycerine consistently failed to produce stable filaments and instead resulted in paste-like material during extrusion. This behaviour reflects glycerine’s high plasticising efficiency, which disrupted the crystallinity and intermolecular hydrogen bonding of the PVA matrix. As a low molecular weight plasticiser (92.09 g/mol) [41], glycerine penetrated the polymer network too effectively, excessively softening the polymer chains. This excessive softening explains the observed paste-like behaviour and lack of molecular entanglement, preventing filament formation. These results aligned with prior studies reporting the destabilising effect of low molecular weight plasticisers when higher concentrations were used, especially in crystalline or semi-crystalline polymers [42]. These observations further corroborate prior findings, which highlighted that increasing the proportion of liquid plasticisers improves flexibility but significantly lowers the glass transition temperature (T_g_) and reduces the physical stability of the amorphous solid dispersions (ASDs) [43]. Glycerine’s strong softening effect on polymer chains underscores the challenge of balancing sufficient plasticisation with maintaining stability. This highlights the critical need for careful optimisation of plasticiser type and concentration to achieve the desired flexibility without decreasing the filament’s structural integrity or thermal stability [44].

The addition of cassava pulp fibres (e.g., P_10_F_5_) significantly altered filament morphology. Fibre-loaded filaments exhibited a darker brown colouration due to the natural colour of cassava pulp and had rougher surfaces with visible voids in cross-sections, as seen in Figure 3 (Figure S1). These voids highlighted incomplete fibre-polymer packing and insufficient adhesion between fibre and PVA matrix in some regions. While the addition of fibres contributed to altered mechanical properties, the resulting surface roughness could hinder smooth feeding in FDM 3D printing and potentially cause filament jamming. Similar to the observations by Kariz et al., who reported that increasing wood content in PLA composites led to surface irregularities, voids, and particle clustering [45]. Such morphological changes increased frictional resistance within the printer nozzle, disrupting the extrusion process [45,46]. In a similar manner, the rougher filament surfaces observed in our formulations may impair feedability and increase the risk of nozzle clogging during printing. To address this potential issue and ensure smooth filament feeding, an additional polymer such as Eudragit^®^ NE 30 D was incorporated into the fibre-loaded filaments. SEM micrograph of P_10_F_5_E_5_ exhibited improved compatibility with the polymer matrix, reducing void formation and achieving smoother cross-sections compared to fibre-only formulations. These findings underscore that the addition of Eudragit^®^ enhanced polymer-fibre adhesion and matrix cohesion, resulting in better structural integrity and reduced heterogeneity. Moreover, the improved surface morphology aligns with the goal of mitigating nozzle clogging by achieving a smoother filament surface, thereby enhancing filament printability. These modifications highlight the importance of balancing polymer interactions to enhance filament quality while ensuring smooth extrusion and reliable 3D printing performance.

The addition of THP at 5% *w*/*w* loading into formulations significantly influenced the surface morphology, as observed in P_10_F_5_E_5_T_5_. SEM micrographs revealed that the drug-loaded filaments exhibited rougher surfaces compared to those without THP (P_10_F_5_E_5_). The increased surface roughness may result from the disruption of the polymer matrix caused by the drug–polymer interaction, which likely affects the packing and uniform dispersion of the matrix. This roughness highlights a potential mixture effect, where the incorporation of THP alters the interaction between other components, creating microstructural irregularities and potentially compromising extrusion smoothness. Interestingly, Eudragit^®^ NE 30 D (P_10_F_5_E_5_T_5_) appeared to play a crucial role in the overall morphology of the filament. The liquid nature of Eudragit^®^ NE 30 D during processing likely contributed to better integration with the polymer matrix and the drug, leading to a more uniform distribution. This may enhance drug–polymer compatibility and help mitigate surface roughness. Although the surface roughness observed in drug-loaded formulations may raise concerns about their use in FDM 3D printing, further studies are necessary to evaluate their mechanical properties and overall printability. These assessments will determine the reliability of filaments in FDM printing for pharmaceutical applications.

### 3.2. Mechanical Properties

**Figure 4:**
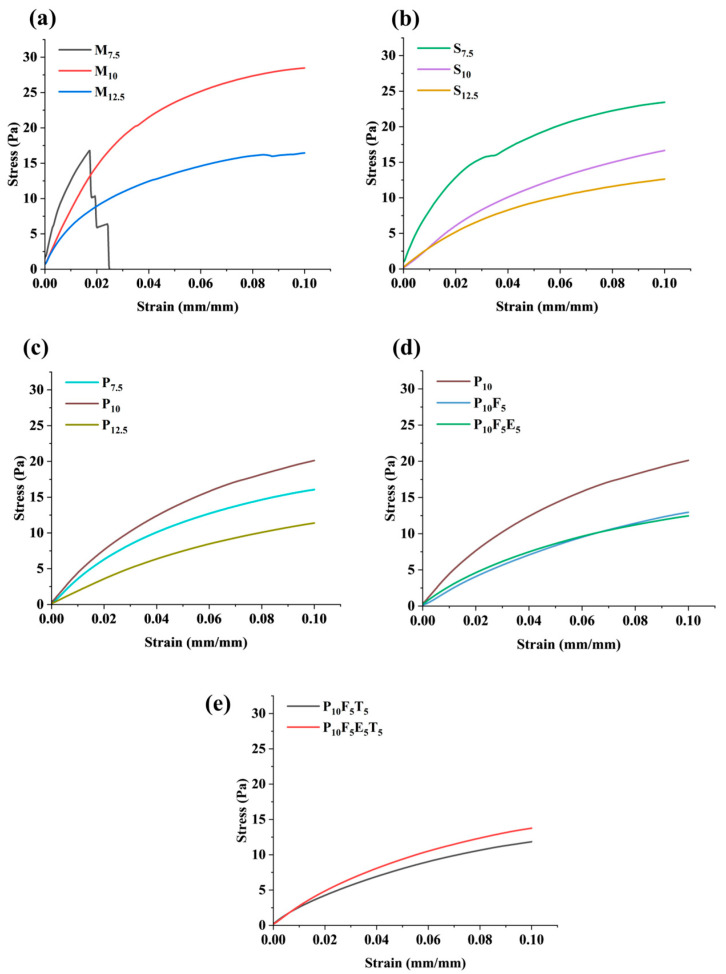
Tensile properties of HME filament with (**a**) mannitol concentration variation; (**b**) sorbitol concentration variation; (**c**) PEG400 concentration variation; (**d**) the influence of short fibres and polymer addition; and (**e**) THP loaded within different formulation compositions.

The mechanical properties of each filament were characterised by its unique stress-strain diagram measured using a tensile test (Figure 4). Most of the filaments showed both elastic and plastic deformation with increasing stress. However, M_7.5_ (i.e., mannitol ratio of 7.5) exhibited its brittleness nature, i.e., breaks without plastic deformation as shown in Figure 4a. A further increase in the ratio of mannitol to 10 and 12.5 produced filaments with plastic deformation properties, but the yield stress significantly decreased from 19.2 ± 1.7 Pa to 7.1 ± 1.3 Pa when the mannitol ratio increased from 10 to 12.5. In the case of sorbitol incorporation into the PVA matrix (Figure 4b), the yield stress of filaments significantly decreased from 14.3 ± 1.0 Pa to 8.4 ± 0.8 Pa when the sorbitol ratio increased from 7.5 to 12.5. This could be due to the plasticising effect of both mannitol and sorbitol, which improves the plastic flexibility but is less rigid, in agreement with previous studies [47,48]. Figure 4c showed an increase in the PEG400 ratio from 7.5 to 10, leading to a significant increase in the yield stress from 12.6 ± 1.5 Pa to 16.3 ± 0.6 Pa. However, as the PEG400 ratio further increased up to 12.5, the yield stress significantly decreased to 10.5 ± 0.7 Pa. This trend is potentially due to the miscibility extent between PVA and PEG400. As a result, a ratio of 10% PEG400 (P_10_) is identified as the optimised level because it provides the best balance between strength and flexibility. Specifically, P_10_ showed a yield stress high enough to maintain filament integrity yet low enough to prevent brittleness and enable smooth feeding. Importantly, P_10_ also exhibited lower variability (±0.6 Pa) compared to other plasticisers, suggesting more consistent and reproducible mechanical behaviour. These mechanical results were consistent with the morphological analysis. P_10_ filaments extruded smoothly and showed homogeneous cross-sections with smooth surfaces, enabling reliable feeding into the FDM printer. In contrast, M_10_ and S_10_ filaments showed slightly rougher surfaces, which could impair feeding. Collectively, these findings explain why P_10_ was chosen as the optimal formulation for further development.

Since P_10_ showed the best tensile performance among the PVA-plasticiser filaments, further investigation on the mechanical properties of P_10_ filaments incorporated with short fibres and polymer was conducted, as shown in Figure 4d. The yield stress significantly decreased from 16.3 ± 0.6 Pa (P_10_) to 11.6 ± 0.5 Pa (P_10_F_5_) upon the addition of 5% short fibre. The lower yield stress observed in P_10_F_5_ suggests increased filament flexibility, which could be advantageous for FDM 3D printing by improving feedability and reducing brittleness-related issues during spool handling and extrusion. No significant difference was noted among filaments when polymer Eudragit^®^ NE 30 D was added. Similarly, no significant difference among filaments was reported when 5% *w*/*w* THP was incorporated into the filament (Figure 4e).

### 3.3. FTIR Spectroscopy Analysis

**Figure 5:**
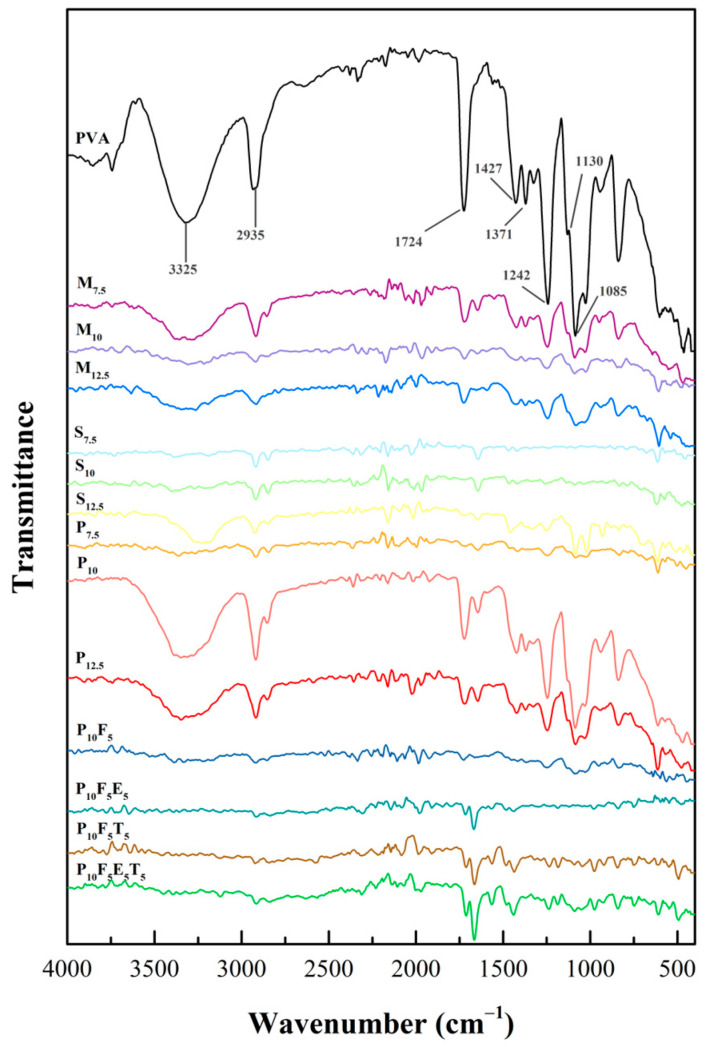
ATR–FTIR spectra of pure PVA and PVA-based composite filaments.

The Fourier transform infrared (FTIR) spectra presented in Figure 5 provided insights into structural composition and chemical interactions within the PVA-based formulations. The spectra for pure PVA displayed characteristic peaks associated with its hydroxyl and acetate groups, which are crucial in defining its intermolecular interactions, crystallinity, and chemical interactions. Consistent with previous studies [49,50,51], the FTIR spectrum of PVA exhibited five different regions corresponding to specific functional group vibrations. The broad band observed in the range of 3500–3250 cm^−1^ was attributed to the O-H stretching. These resulted from the intermolecular and intramolecular hydrogen bonding within the polymeric structure of PVA, indicating its hydrophilic nature and extensive hydrogen-bonding network. A peak near 2935 cm^−1^ corresponded to the C-H stretching of alkyl groups, while the 1724 cm^−1^ peak arose from C=O stretching vibrations associated with residual acetate groups from incomplete hydrolysis during PVA synthesis [49,52,53]. In the fingerprint region, the peak at 1130 cm^−1^ correlated with the degree of crystallinity, attributed to C-O and symmetric C-C stretching and reflected the organised crystalline portions of the polymer [54,55]. Additionally, the peak near 1085 cm^−1^ corresponded to C-O-C bonding, confirming the structural integrity of PVA. In addition, the peaks at 1427 cm^−1^ and 1371 cm^−1^ were attributed to the -CH_2_ twisting vibration and C-H/O-H bending, respectively. These peaks provided further confirmation of the structural features of PVA, including the vibrational modes of its alkyl groups and hydroxyl functionalities. Their consistent presence across all formulations reinforced the structural stability of PVA under the experimental conditions.

When plasticisers such as mannitol, sorbitol, and PEG400 were added, significant changes were observed in the O-H stretching region, indicating their impact on hydrogen bonding within the PVA matrix. Mannitol and sorbitol, with their smaller molecular sizes (182.17 g/mol) [41], formed extensive hydrogen bonds with the OH groups of PVA. This increased bonding reduces intermolecular rigidity and induces rearrangement of polymer chains, thereby enhancing the chain’s mobility and flexibility of the filaments [56], as observed in the mechanical data where yield stress decreased with increasing concentrations of mannitol and sorbitol (Figure 4a,b). Conversely, PEG400, with a higher molecular weight (~400 g/mol) [41], showed a weaker interaction with PVA’s hydroxyl groups, as evidenced by the presence of the O-H peak in the FTIR spectra of PEG400-based formulations (P_7.5_, P_10_, and P_12.5_). The larger molecular size of PEG400 compared to mannitol and sorbitol likely contributed to a reduced density of hydrogen bonding interactions, limiting its ability to penetrate and disrupt the PVA matrix as effectively as mannitol and sorbitol, as its bulkier structure hindered close packing within the polymer matrix. However, this partial bonding created a balance between rigidity and flexibility in the PEG-based filaments, as reflected in their mechanical properties. Among the PEG formulations, P_10_ demonstrated superior mechanical properties compared to P_7.5_ and P_12.5_, with optimal yield stress (Figure 4c). This lower interaction density explains why PEG-based formulations retained some rigidity while still achieving superior flexibility at the 10% *w*/*w* ratio. At 12.5% PEG concentration (P_12.5_), the mechanical performance declined, likely due to excessive plasticisation disrupting the matrix’s structural integrity. These results underscore the importance of molecular size and concentration in optimising mechanical properties, with P_10_ being the most suitable formulation due to its superior flexibility and tensile performance.

Further structural and mechanical modifications were observed with the addition of short fibres, such as cassava pulp waste, at a 5% concentration (P_10_F_5_). FTIR spectra revealed the disappearance of the O-H peak, reflecting hydrogen bonding between the cellulose fibres and the PVA–PEG matrix. These interfacial hydrogen bonds can restrict local chain mobility while allowing more flexible regions away from the fibre surface. While the addition of fibres is generally expected to enhance the structural rigidity of the material, the mechanical testing results showed a decrease in yield stress when fibres were incorporated (as seen in Figure 4d), indicating an increase in flexibility. This reduction in yield stress suggests that the fibres reduced the stiffness of the material, allowing it to deform more easily under applied stress. The presence of fibres likely disrupted the compactness of the polymer network, creating a less dense and more flexible matrix, while hydrogen bonding and van der Waals interactions at the fibre–polymer interface contributed to overall structural integrity. This less dense network of interactions would result in a material that is more capable of flexing and bending without breaking, thereby reducing the yield stress and enhancing the material’s flexibility. In this case, the fibres contributed to a more flexible, less rigid filament despite their typical role in reinforcing the matrix. The reduction in yield stress, therefore, directly correlates with increased flexibility, allowing the material to undergo more deformation before breaking.

The incorporation of THP as a model drug into the formulations introduced additional spectral changes. A new peak emerged around ~1650 cm^−1^ in the P_10_F_5_T_5_ formulation compared to P_10_F_5_, corresponding to the presence of THP (C=O stretching amide) [57]. However, the addition of Eudragit^®^ NE 30 D caused these peaks to become less pronounced due to overlapping signals from the polymers. This suggests that while THP was successfully incorporated into the filament, its interaction with PVA and PEG was minimal. This is an important observation for drug delivery applications, as it indicates that the polymer matrix did not significantly alter the properties of the drug during the filament formation process. The mechanical properties of the filaments were also not significantly affected by the addition of THP. The FTIR spectra showed no substantial changes in the O-H peak at around 3200–3500 cm^−1^, confirming that there were no significant interactions between the drug and the polymer matrix. This result was supported by the mechanical testing data in Section 3.2, which showed that the tensile properties of the drug-loaded filaments remained relatively unchanged after the incorporation of the drug. This finding suggests that the presence of THP did not adversely affect the filament’s mechanical integrity, making it a suitable candidate for 3D-printed drug delivery applications.

### 3.4. Thermal Stability Analysis

**Figure 6:**
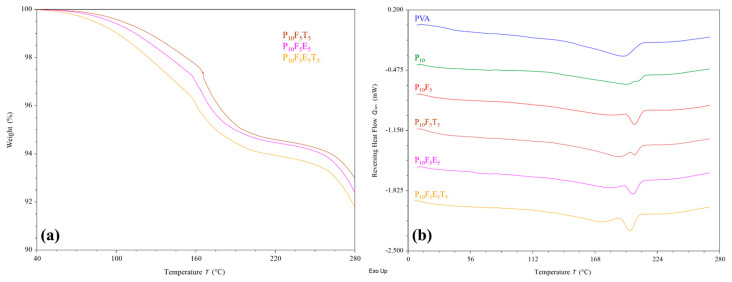
(**a**) TGA and (**b**) DSC thermograms of PVA-based filaments incorporated with plasticiser, fibre, polymer, and drug. Raw PVA powder was used as a control in comparison to the formulated filaments in DSC.

Thermal analyses were conducted to assess the thermal stability of selected formulated filaments in response to the applied processing temperature. The results from TGA (Figure 6a) reflected the thermal stability of all selected filaments at their extrusion and printing temperature, i.e., 195–210 °C and 200 °C, respectively, where less than 10% weight reduction was identified. This further proves that the brown colouration observed in the filaments was due to the incorporation of brownish cassava pulp fibres rather than material degradation upon heating.

As shown in Figure 6b, DSC analysis revealed an upshifting melting peak of PVA upon the addition of PEG400 plasticiser, similar to previous observations [44,58]. The peak broadening of PVA could be attributed to the interaction between PVA and PEG, as discussed in FTIR analysis. Interestingly, the further incorporation of fibres (P_10_F_5_) reduced the peak broadening and increased the enthalpy of the shifted melting peak. This suggests that although fibres interact with the PVA–PEG matrix, they help maintain a more ordered crystalline structure locally while still enhancing flexibility, as confirmed by mechanical testing. In the case of introducing additional polymer (Eudragit^®^ NE 30 D) and THP to the PVA–PEG-fibre matrix, a general melting peak broadening and enthalpy reduction were identified. However, such changes did not reflect a significant effect on the chemical interaction as determined in the FTIR analysis and the mechanical properties of the filaments. Overall, the finding of this study indicates that the addition of fibres, plasticisers, polymer, and drug did not compromise the thermal stability of the filaments, supporting their suitability for FDM 3D printing applications.

### 3.5. Drug Loading Content

The recovery percentages for THP-loaded filaments were 109.24 ± 4.20% and 102.20 ± 1.14% for P_10_F_5_T_5_ and P_10_F_5_E_5_T_5_ formulations, respectively. Both values fall within the acceptable range specified by the United States Pharmacopeia-National Formulary (USP-NF), which requires a recovery of not less than 90.0% (NLT) and not more than 110.0% (NMT) of the labelled amount of anhydrous theophylline formulation [59]. Among the tested formulations, P_10_F_5_T_5_ exhibited the highest standard deviation (SD) of 4.20%, indicating greater variability in drug distribution within the filament. This variability may be attributed to the incomplete integration of cassava pulp fibres within the polymer matrix, leading to surface roughness and voids, as observed in SEM micrographs. These structural irregularities likely contributed to non-uniform drug dispersion along the filament. In contrast, the formulation containing Eudragit^®^ NE 30 D (P_10_F_5_E_5_T_5_) demonstrated a lower SD, suggesting improved drug distribution uniformity. The inclusion of Eudragit^®^ enhanced fibre-polymer cohesion, mitigating phase separation and reducing content variability. These findings underscore the importance of polymer additives in promoting matrix homogeneity and ensuring consistent drug loading. Based on these findings, P_10_F_5_E_5_T_5_ was selected for further printability and drug release studies due to its superior homogeneity and consistent drug loading.

### 3.6. Effect of Microstructural Design on Drug Release Behaviour

**Figure 7:**
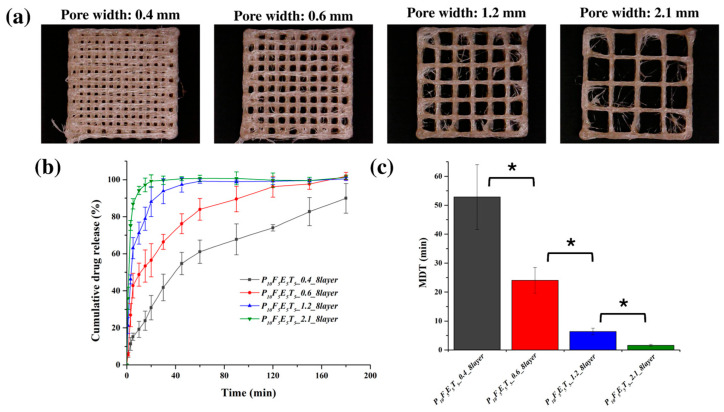
(**a**) Representative images of the 3D-printed constructs with varying pore widths, (**b**) Drug release profiles of constructs with different pore widths in PBS pH 7.4 at 37 °C, and (**c**) MDT values of the constructs with varying pore widths (*n* = 3). An asterisk symbol (*) indicates significant differences in MDT values between constructs (*p* < 0.05).

The morphology of the 3D-printed constructs, as observed in Figure 7a, demonstrated a well-defined porous structure, closely resembling the original CAD design, particularly in the case of the larger pore structures. The accuracy of pore replication indicates a well-controlled printing process, which is essential for maintaining consistent porosity and ensuring predictable drug release kinetics. Notably, the incorporation of cassava fibres within the construct was evident in the fibre-like features seen in the microscopic images. These fibres may contribute to the overall mechanical properties of the printed material while also influencing drug release behaviour through their interaction with the polymer matrix and drug molecules.

The presence of larger pores played a critical role in modulating the drug release kinetics. As confirmed in Figure 7b, the release kinetics exhibited a clear dependency on pore width, with the constructs featuring wider pores demonstrating faster drug release rates. Specifically, constructs with the largest pore width (2.1 mm) exhibited the most rapid drug release, with approximately 99.66 ± 2.28 % of the drug released within the first 30 min, while the narrowest pore width (0.4 mm) resulted in the slowest release, achieving only 41.81 ± 7.16 % of drug release over the same period. This trend aligns with the hypothesis that increased porosity leads to a greater surface area-to-volume (SA/V) ratio (Table 6) and shorter diffusion pathways, facilitating more efficient drug release. The mean dissolution time (MDT) values further corroborated this observation (Figure 7c), with the construct featuring a pore width of 2.1 mm having the lowest MDT (1.64 ± 0.31 min), indicating a significantly faster release rate compared to the construct with a 0.4 mm pore width (52.82 ± 11.22 min, *p* < 0.05).

**Figure 8:**
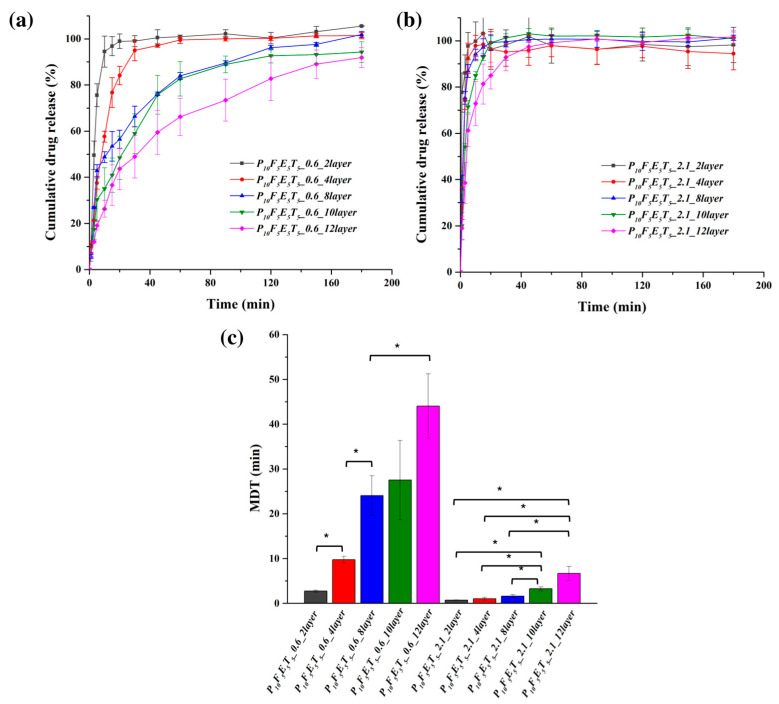
Drug release profiles of constructs with different layer numbers for a fixed pore width of (**a**) 0.6 mm and (**b**) 2.1 mm, and (**c**) MDT values for constructs with varying layer numbers (*n* = 3). An asterisk symbol (*) indicates significant differences in MDT values between constructs (*p* < 0.05).

The effect of construct thickness (layer number) on drug release was also assessed using constructs with fixed pore widths of 0.6 mm and 2.1 mm, while varying the number of layers from 2 to 12 (Figure 8a,b). The results indicate that increasing the number of layers led to a more sustained release profile, particularly in the case of the 0.6 mm pore width samples. Constructs with only two layers (P_10_F_5_E_5_T_5__0.6_2layer) exhibited the fastest drug release, with nearly 100% of the drug released within the first 30 min, whereas constructs with 12 layers (P_10_F_5_E_5_T_5__0.6_12layer) showed a more prolonged release profile with 48.92 ± 9.23% and 91.88 ± 4.31% of drug release in 30 min and 3 h, respectively.

In contrast, for the constructs with a pore width of 2.1 mm, drug release remained rapid across all layer numbers but demonstrated a slight trend toward sustained release as the number of layers increased. Constructs with two layers (P_10_F_5_E_5_T_5__2.1_2layer) exhibited almost immediate drug release, with over 85 % in 3 min. However, as the layer number increased, a subtle delay in drug release was observed. Specifically, constructs with 12 layers (P_10_F_5_E_5_T_5__2.1_12layer) exhibited a more prolonged release, with approximately 38.52 ± 8.91 % released within 3 min and complete drug release by 1 h. The MDT values (Figure 8c) further emphasise this trend, with constructs featuring 10 and 12 layers showing significantly higher MDT values compared to those with 2–8 layers, confirming that additional thickness at large pore width could still slow down the drug release in a measurable manner. Specifically, constructs with two layers had significantly lower MDT values (0.75 ± 0.06 min for P_10_F_5_E_5_T_5__2.1_2layer) than those with 12 layers (6.70 ± 1.59 min for P_10_F_5_E_5_T_5__2.1_12layer, *p* < 0.05).

These findings indicate that while increasing the number of layers led to a more sustained release profile, the effect was not as pronounced as in constructs with narrower pores (e.g., 0.6 mm). In the 2.1 mm pore width constructs, rapid release occurred at different layer numbers, underscoring the dominant role of high porosity in facilitating faster dissolution despite increased thickness. Notably, beyond 10 layers, the release rate plateaued as the MDT values of P_10_F_5_E_5_T_5__2.1_10layer and P_10_F_5_E_5_T_5__2.1_12layer were not significantly different, thus suggesting that further increasing construct thickness does not significantly alter drug diffusion kinetics at this pore width.

A comparison with our previous work [37] using PCL as the polymer matrix instead of PVA highlights key differences in release behaviour. In the PCL-based drug delivery systems reported by Zhang et al. (2021) [37], the drug release was described as following a diffusion mechanism, which is consistent with the insoluble and non-swellable nature of PCL in aqueous media. In such systems, drug transport is predominantly controlled by diffusion through a stable hydrophobic matrix, resulting in more sustained release profiles. Notably, however, no kinetic fitting was performed in that study; rather, the diffusion-type behaviour was inferred from the physicochemical properties of PCL and the observed release patterns. In contrast, in this study, the present PVA-based constructs not only displayed faster release due to the hydrophilic nature of PVA but also allowed for a more detailed kinetic analysis through model fitting. According to the kinetic fitting results presented in Table 7, the release profiles of the constructs with narrower pores (0.6 mm) were well described by both the Higuchi and Korsmeyer–Peppas models, with coefficients of determination (R^2^) exceeding 0.95 in most cases, suggesting that the release behaviour was well described by diffusion-based processes. The n values obtained from the Korsmeyer–Peppas model ranged between 0.42 and 0.66. Values close to 0.45 (<0.45) are indicative of hindered or Fickian diffusion, whereas higher values (>0.45) fall within the anomalous transport regime [60,61]. This indicates that drug release in the 0.6 mm constructs involved a mixed mechanism, where Fickian diffusion dominated in thinner constructs but swelling of the PVA matrix became increasingly relevant in thicker constructs, leading to more sustained release profiles. Conversely, constructs with larger pores (2.1 mm) exhibited much lower n values (0.08–0.40), which are characteristic of hindered Fickian diffusion. In these highly porous constructs, drug release was governed predominantly by fast diffusion through the interconnected channels, with negligible contribution from polymer relaxation or swelling. The almost complete drug release within 30 min further supports that these constructs display an immediate release behaviour, in contrast to the more sustained release observed in narrower pore systems.

Taken together, these observations suggest that drug release from 3D-printed constructs is governed by a dual mechanism in which both structural (pore size, thickness) and material-related (polymer hydrophilicity and fibre incorporation) parameters act synergistically. At low porosity, release follows a diffusion-swelling interplay consistent with the Higuchi model and anomalous transport, while at high porosity, rapid Fickian diffusion dominates regardless of construct thickness. This mechanistic understanding is crucial for the rational design of tailored drug delivery systems, where the interplay between pore architecture and polymer properties can be tuned to achieve desired release profiles.

## 4. Conclusions

This study highlights the critical role of both formulation and structural design in determining the performance of PVA-based filaments for pharmaceutical FDM 3D printing, while also demonstrating, for the first time, the potential of cassava pulp-derived fibres as sustainable additives for eco-friendly filament development. Compared with previous studies on natural fibres, which have focused mainly on hydrophobic polymers for non-pharmaceutical applications, our work establishes cassava fibres as an effective mechanical modifier in hydrophilic PVA systems, thereby addressing an important gap in the state of knowledge.

Plasticiser selection strongly influenced filament quality: PEG400, mannitol, and sorbitol enabled successful extrusion, while glycerine disrupted the polymer network. Mechanical testing showed that plasticiser concentration determined the balance between flexibility and rigidity, with PEG400 at 10% *w/w* (P_10_) providing optimal mechanical properties (yield stress of 16.3 ± 0.6 Pa). Fibre incorporation further improved flexibility (yield stress reduced to 11.6 ± 0.5 Pa) but increased surface roughness, which could hinder feedability. Drug loading (5% *w/w* theophylline) altered surface features without compromising integrity, while Eudragit^®^ NE 30 D improved matrix uniformity, reducing drug content variability (SD from 4.20% to 1.14%).

The optimised formulation (P_10_F_5_E_5_T_5_) was successfully employed in FDM printing and exhibited geometry-dependent release behaviour. Constructs with wider pores (2.1 mm) achieved nearly complete release within 30 min, whereas narrower pores and thicker designs produced sustained release governed increasingly by polymer swelling. This confirms that drug release kinetics can be tuned through geometry without altering formulation, adding a novel control strategy to pharmaceutical FDM.

Overall, this work advances the field by integrating sustainable raw materials, optimised formulation strategies, and geometry-driven release control into a single framework for personalised medicine. The dual-release capability of the developed constructs highlights their potential to meet both immediate and sustained therapeutic needs. Looking forward, further research could explore fibres from other natural sources (e.g., bamboo, hemp, pineapple) to compare reinforcement and release behaviours, while applying X-ray diffraction (XRD) would provide deeper insight into how fibre incorporation influences crystallinity, structural organisation, and thermal stability.

## Figures and Tables

**Table 1:** Materials used in the study, their type, origin, and functional role in pharmaceutical formulation.

Material (Class/Compound)	Origin/Supplier	Role in Formulation
Cassava fibres – natural cellulose fibres derived from cassava pulp waste	Agro-industrial by-product (Thailand)	Mechanical modifier additive
Polyvinyl alcohol (PVA) – Mowiol^®^ 4-88, partially hydrolysed PVA, MW ~31,000 g/mol	Sigma-Aldrich^®^ (St. Louis, MO, USA)	Matrix-former, primary filament base
Glycerin – polyol (sugar alcohol)	Union Science Co., Ltd. (Chiang Mai, Thailand)	Plasticiser
Polyethylene glycol (PEG 400) – polyether		
Sorbitol – polyol (sugar alcohol)		
Mannitol – polyol (sugar alcohol)	MySkinRecipes (Bangkok, Thailand)	
Calcium stearate – fatty acid salt	MySkinRecipes (Bangkok, Thailand)	Lubricant and stabiliser
Poly(ethyl acrylate, methyl methacrylate) with 1.5% (nonoxynol) 2:1 (aqueous dispersion) – Eudragit^®^ NE 30 D	Evonik Industries AG (Essen, Germany)	Functional excipient (processing aid)
Theophylline anhydrous (C_7_H_8_N_4_O_2_, MW 180.16 g/mol) – xanthine derivative	Acros Organics™ (Geel, Belgium)	API (for asthma and chronic obstructive pulmonary disease)

**Table 2:** Composition of PVA–plasticiser filament formulations (% *w/w*).

Formulation Code	PVA	Mannitol	Glycerine	Sorbitol	PEG400
M_7.5_	92.5	7.5	-	-	-
M_10_	90.0	10.0	-	-	-
M_12.5_	87.5	12.5	-	-	-
G_7.5_	92.5	-	7.5	-	-
G_10_	90.0	-	10.0	-	-
G_12.5_	87.5	-	12.5	-	-
S_7.5_	92.5	-	-	7.5	-
S_10_	90.0	-	-	10.0	-
S_12.5_	87.5	-	-	12.5	-
P_7.5_	92.5	-	-	-	7.5
P_10_	90.0	-	-	-	10.0
P_12.5_	87.5	-	-	-	12.5

**Table 3:** Composition of short fibre-loaded filament formulations (% *w/w*).

Formulation Code	PVA	PEG400	Short Fibre	Eudragit^®^ NE 30 D	Calcium Stearate	THP
P_10_F_5_	85.0	10.0	5.0	-	-	-
P_10_F_5_E_5_	79.0	10.0	5.0	5.0	1.0	-
P_10_F_5_T_5_	79.0	10.0	5.0	-	1.0	5.0
P_10_F_5_E_5_T_5_	74.0	10.0	5.0	5.0	1.0	5.0

**Table 4:** Optimised extrusion parameters for filament production.

Formulation Code	Pre-Heat Temperature (°C)	Extrusion Temperature (°C)	Screw Speed (rpm)	Conveyor Belt Speed (mm/s)
M_7.5_	195	210	7.5	3.85
M_10_	195	210	7.5	3.85
M_12.5_	195	200	7.5	3.33
G_7.5_	NA	NA	NA	NA
G_10_	NA	NA	NA	NA
G_12.5_	NA	NA	NA	NA
S_7.5_	195	210	6.7	2.20
S_10_	195	210	6.7	2.00
S_12.5_	190	190	8.6	0.67
P_7.5_	195	210	6.7	2.22
P_10_	195	210	7.5	2.04
P_12.5_	195	195	8.6	0.67
P_10_F_5_	195	200	7.5	0.78
P_10_F_5_E_5_	190	195	7.5	2.08
P_10_F_5_T_5_	195	200	7.5	2.33
P_10_F_5_E_5_T_5_	195	200	7.5	2.17

Note: NA (Not applicable) means the filaments could not be obtained through the single-screw, hot-melt extruder.

**Table 5:** CAD parameters of the 3D constructs used in the drug release studies.

Design Type	Pore Width (mm)	3D Construct Width (mm)	3D Construct Length (mm)	3D Construct Thickness (mm)
**Pore width (0.4–2.1 mm)**				
P_10_F_5_E_5_T_5__0.4_8layer	0.4	10	10	1.80
P_10_F_5_E_5_T_5__0.6_8layer	0.6	10	10	1.80
P_10_F_5_E_5_T_5__1.2_8layer	1.2	10	10	1.80
P_10_F_5_E_5_T_5__2.1_8layer	2.1	10	10	1.80
**Layer number (2–12 layers with 0.6- and 2.1-mm pore width)**				
P_10_F_5_E_5_T_5__0.6_2layer	0.6	10	10	0.60
P_10_F_5_E_5_T_5__0.6_4layer	0.6	10	10	1.00
P_10_F_5_E_5_T_5__0.6_8layer	0.6	10	10	1.80
P_10_F_5_E_5_T_5__0.6_10layer	0.6	10	10	2.20
P_10_F_5_E_5_T_5__0.6_12layer	0.6	10	10	2.60
P_10_F_5_E_5_T_5__2.1_2layer	2.1	10	10	0.60
P_10_F_5_E_5_T_5__2.1_4layer	2.1	10	10	1.00
P_10_F_5_E_5_T_5__2.1_8layer	2.1	10	10	1.80
P_10_F_5_E_5_T_5__2.1_10layer	2.1	10	10	2.20
P_10_F_5_E_5_T_5__2.1_12layer	2.1	10	10	2.60

**Table 6:** Effect of pore width and layer number on the calculated SA/V ratios of 3D-printed constructs using a CAD model.

Design Type	Pore Width (mm)	Surface Area (mm^2^)	Solid Volume (mm^3^)	*SA*/*V* Ratio (mm^−1^)
**Pore width (0.4–2.1 mm)**				
P_10_F_5_E_5_T_5__0.4_8layer	0.4	831.36	113.23	7.34
P_10_F_5_E_5_T_5__0.6_8layer	0.6	790.72	100.80	7.84
P_10_F_5_E_5_T_5__1.2_8layer	1.2	580.80	68.89	8.43
P_10_F_5_E_5_T_5__2.1_8layer	2.1	440.78	51.46	8.57
**Layer number (2–12 layers with 0.6- and 2.1-mm pore width)**				
P_10_F_5_E_5_T_5__0.6_2layer	0.6	242.44	27.33	8.87
P_10_F_5_E_5_T_5__0.6_4layer	0.6	425.20	51.82	8.21
P_10_F_5_E_5_T_5__0.6_8layer	0.6	790.72	100.80	7.84
P_10_F_5_E_5_T_5__0.6_10layer	0.6	973.49	125.30	7.77
P_10_F_5_E_5_T_5__0.6_12layer	0.6	1156.26	149.79	7.72
P_10_F_5_E_5_T_5__2.1_2layer	2.1	127.82	13.82	9.25
P_10_F_5_E_5_T_5__2.1_4layer	2.1	232.14	26.37	8.80
P_10_F_5_E_5_T_5__2.1_8layer	2.1	440.78	51.46	8.57
P_10_F_5_E_5_T_5__2.1_10layer	2.1	545.11	64.02	8.51
P_10_F_5_E_5_T_5__2.1_12layer	2.1	649.22	76.57	8.48

**Table 7:** Release kinetic parameters of 3D-printed constructs fitted to various mathematical models.

Design Type	Kinetic Model				
	Higuchi Matrix		Korsmeyer–Peppas		
	R^2^	*k_H_* (min^1/2^)	R^2^	*k_K_* (min^−n^)	n
P_10_F_5_E_5_T_5__0.6_2layer	0.8355	23.35	0.9257	30.92	0.42
P_10_F_5_E_5_T_5__0.6_4layer	0.9913	22.42	0.9938	12.30	0.66
P_10_F_5_E_5_T_5__0.6_8layer	0.8600	13.58	0.9364	16.64	0.43
P_10_F_5_E_5_T_5__0.6_10layer	0.9534	11.37	0.9764	10.90	0.50
P_10_F_5_E_5_T_5__0.6_12layer	0.9890	10.61	0.9973	6.40	0.64
P_10_F_5_E_5_T_5__2.1_2layer	0.6501	5.87	0.8819	80.46	0.08
P_10_F_5_E_5_T_5__2.1_4layer	0.6509	16.41	0.8616	50.41	0.25
P_10_F_5_E_5_T_5__2.1_8layer	0.7441	15.59	0.9100	51.51	0.24
P_10_F_5_E_5_T_5__2.1_10layer	0.8980	21.13	0.9616	33.55	0.38
P_10_F_5_E_5_T_5__2.1_12layer	0.9199	18.85	0.9681	26.94	0.40

## Data Availability

The dataset is available upon request from the authors.

## Supplementary Materials

The following supporting information can be downloaded at: https://www.mdpi.com/article/10.3390/polym17182502/s1, Figure S1: Macroscopic images of M_10_, S_10_, P_10_, P_10_F_5_, P_10_F_5_T_5_, P_10_F_5_E_5_, and P_10_F_5_E_5_T_5_ filaments.
