# Supplementary material for: Formulation and Structural Optimisation of PVA-Fibre Biopolymer Composites for 3D Printing in Drug Delivery Applications
Source: Polymers (Basel). 2025 Sep 16;17(18):2502. doi: 10.3390/polym17182502 (PMC12473208; doi:10.3390/polym17182502)
Supplement: Supplementary file 1 [file polymers-17-02502-s001.zip › polymers-3833969-supplementary.pdf]

polymers

Supplementary Materials: Formulation and Structural Optimisation of PVA-Fibre Biopolymer Composites for 3D Printing in Drug Delivery Applications

Pattaraporn Panraksa [1], Pensak Jantrawut [1,*], Xin Yi Teoh [2,3], Krit Sengtakdaed [1], Ploynapat Pornngam [1], Tanpong Chaiwarit [1], Takron Chantadee [1], Kittisak Jantanasakulwong [4], Suruk Udomsom [5], and Bin Zhang [6,*]

1 Department of Pharmaceutical Sciences, Faculty of Pharmacy, Chiang Mai University, Chiang Mai 50200, Thailand; pattaraporn.pan@cmu.ac.th (P.P.); krit.sengtakdaed@gmail.com (K.S.); ploynapat2001@gmail.com (P.P.); tanpong.ch@cmu.ac.th (T.C.); takron.chantadee@cmu.ac.th (T.C.)
2 School of Pharmacy, Monash University Malaysia, Subang Jaya 47500, Selangor Darul Ehsan, Malaysia; txy1807@gmail.com (X.Y.T.)
3 School of Pharmacy, University College London, London WC1N 1AX, UK
4 Division of Packaging Technology, School of Agro-Industry, Faculty of Agro-Industry, Chiang Mai University, Chiang Mai 50100, Thailand; jantanasakulwong.k@gmail.com (K.J.)
5 Biomedical Engineering Institute, Chiang Mai University, Chiang Mai 50200, Thailand; suruk_u@cmu.ac.th (S.U.)
6 Department of Mechanical and Aerospace Engineering, Brunel University London, UB8 3PH, UK
* Correspondence: pensak.j@cmu.ac.th (P.J.); bin.zhang@brunel.ac.uk (B.Z.); Tel.: +66-539-443-09 (P.J.); +44-(0)1895-268573 (B.Z.)

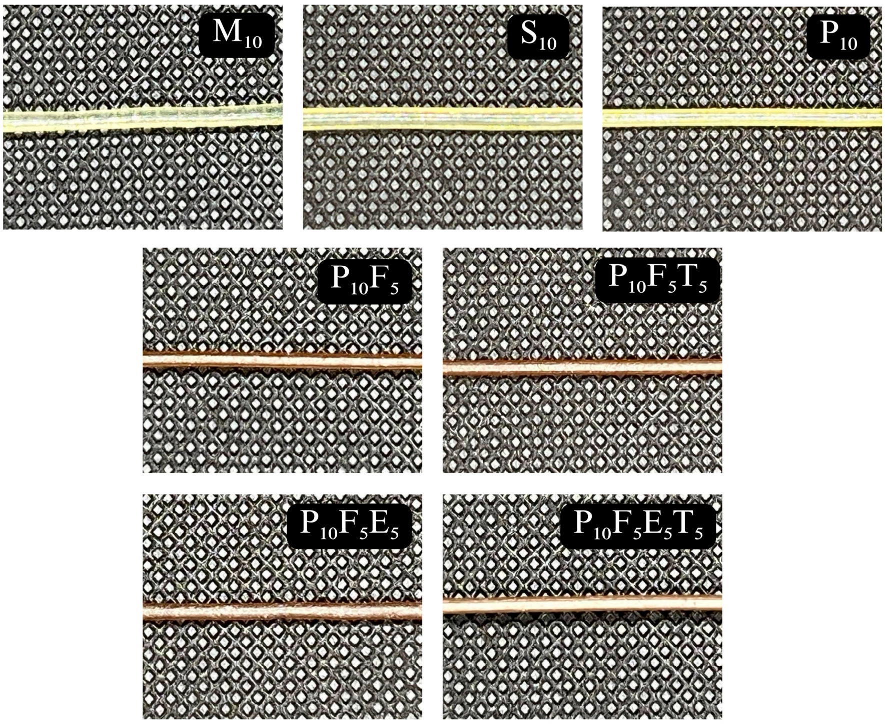

Figure S1. Macroscopic images of M_{10}, S_{10}, P_{10}, $P_{10}F_5$, $P_{10}F_5T_5$, $P_{10}F_5E_5$, and $P_{10}F_5E_5T_5$ filaments.

 https://doi.org/10.3390/polym17182502
